# Application Prospects of Mesenchymal Stem Cell Therapy for Bronchopulmonary Dysplasia and the Challenges Encountered

**DOI:** 10.1155/2021/9983664

**Published:** 2021-05-03

**Authors:** Yajie Tong, Jingye Zuo, Dongmei Yue

**Affiliations:** Department of Pediatrics, Shengjing Hospital of China Medical University, No. 36, Sanhao Street, Heping District, Shenyang, 110004 Liaoning, China

## Abstract

Bronchopulmonary dysplasia (BPD) is a common chronic lung disease in premature babies, especially affecting those with very low or extremely low birth weights. Survivors experience adverse lung and neurological defects including cognitive dysfunction. This impacts the prognosis of children with BPD and may result in developmental delays. The currently available options for the treatment of BPD are limited owing to low efficacy or several side effects; therefore, there is a lack of effective treatments for BPD. The treatment for BPD must help in the repair of damaged lung tissue and promote further growth of the lung tissue. In recent years, the emergence of stem cell therapy, especially mesenchymal stem cell (MSC) therapy, has improved the treatment of BPD to a great extent. This article briefly reviews the advantages, research progress, and challenges faced with the use of MSCs in the treatment of BPD. Stem cell therapy is beneficial as it repairs damaged tissues by reducing inflammation, fibrosis, and by acting against oxidative stress damage. Experimental trials have also proven that MSCs provide a promising avenue for BPD treatment. However, there are challenges such as the possibility of MSCs contributing to tumorous growths, the presence of heterogeneous cell populations resulting in variable efficacy, and the ethical considerations regarding the use of this treatment in humans. Therefore, more research must be conducted to determine whether MSC therapy can be approved as a treatment option for BPD.

## 1. Introduction

Bronchopulmonary dysplasia (BPD) is a common chronic lung disease in premature infants, especially in very low birth weight (VLBW) and extremely low birth weight (ELBW) infants. Epidemiological investigations show that the incidence of BPD in VLBW and ELBW infants is 30%–40% and 54.1%, respectively [1]. In the early postnatal period of premature infants, the cells in lung in the late canalicular or early saccular stage are in a highly proliferative state. Therefore, these preterm infants lack effective alveolar gas exchange. BPD is a result of lung injury and abnormal lung repair, characterized by a decrease in the number of alveoli, abnormal morphology, uneven distribution of ventilation, and abnormal development of the pulmonary vascular system [2, 3]. Hyperoxia, mechanical ventilation, inflammation, and oxidative stress are the main causes of disease. And the antioxidant capacity of preterm infants is insufficient, which facilitates oxidative stress damage as hyperoxia produces more oxygen free radicals which then exceed the antioxidant power of these infants. Prematurity and low birth weight are the most important factors in the development of BPD, and other factors such as intrauterine growth restriction (IUGR), chorioamnionitis, race, or ethnicity also contribute to its process.

Survivors of BPD often suffer from sequelae such as airway hyperresponsiveness and abnormal pulmonary function [4]. Additionally, some survivors may have adverse neurological outcomes such as motor and cognitive dysfunction, which significantly affects their quality of life [5]. Currently, there is a lack of effective treatment for BPD. Although glucocorticoids, vitamin A, and caffeine have specific preventive and therapeutic effects on BPD, they are limited by low long-term efficacy or severe adverse reactions [6, 7]. The treatment for BPD is not only to repair the lung injury but also to promote lung growth. Stem cell therapy, especially using mesenchymal stromal cells (MSCs), has advanced BPD therapeutic effect. This article briefly reviews the advantages, progress, and challenges of MSCs in the treatment of BPD.

## 2. Basic Characteristics and Mechanisms of MSCs

MSCs refer to mesenchymal stromal cells, and they are not true “stem” cells, since their main functionality is not multipotency in vivo. MSCs may be sourced from embryonic, perinatal (umbilical cord, placenta, amniotic membrane, umbilical cord blood, and chorion), and adult tissues (bone marrow, fat, skin, dental pulp, synovium, and liver) [8].

They have the following basic characteristics [9, 10]: (1) Under standard culture conditions, bone marrow MSCs must be attached to plastic culture vessels when cultured in tissue culture bottles. (2) MSCs must express the cluster of differentiation (CD) markers CD73, CD90, and CD105 on their cell surface. (3) Bone marrow MSCs should lack hematopoietic markers such as CD45 (white blood cells), CD34 (hematopoietic progenitor cells), CD14 or CD11b (monocytes/macrophages), CD19 or CD79a (B cells), or human leukocyte antigen DR (HLA-DR) which is a major histocompatibility complex class II receptor. (4) They can differentiate into adipocytes, osteocytes, and chondrocytes.

1. Homing process

Homing is a crucial part of the healing process facilitated by MSCs. Studies have confirmed that MSCs preferentially enter injured sites [11], although the migration of MSCs to these sites is not fully understood. It is likely that they respond to chemokines and migrate under the stimulation of these signals from inflammatory cells [11, 12]. Cell adhesion molecules expressed by MSCs interact with these cytokines and reach the target organs through local or systemic circulatory migration and endothelial cell transport [13]. Once MSCs reach the target tissue injury area, they can play their therapeutic role [14].

(2) Paracrine production

Many studies show therapeutic efficacy of MSCs is through paracrine actions rather than cell differentiation or cell–cell contact. In vitro and in vivo studies have shown that MSC-conditioned media, that is, cell-free media which contains metabolites obtained from MSCs, can protect alveolar epithelial cells and pulmonary microvascular endothelial cells from oxidative stress when cultured for 24 hours [15, 16]. This is because MSCs can secrete growth factors, chemokines that induce cell proliferation, angiogenesis, antiapoptosis, antioxidation, and antifibrosis components that can be detected in MSC-conditioned media [17–20]. A study confirmed that MSC-conditioned medium reduced the apoptosis of AEC-IIs exposed to hyperoxia [19]. Another study indicated that the overexpression of angiopoietin-1 in MSCs promoted recovery of lipopolysaccharide-induced lung tissue injury, suggesting that secretory factors contribute to the healing process [21]. Similarly, when treated with MSC-conditioned medium alone, lung repair in mice with hyperoxia-induced lung injury was also observed in the absence of cells [20]. MSC transplantation can decrease the levels of classical proinflammatory cytokines, including Interleukin(IL)-1*β* and IL-6, macrophage inflammatory protein- (MIP-) 1*α*, tumor necrosis factor- (TNF-) *α*, and transforming growth factor- (TGF-) *β*1, and increase the levels of central growth factors and anti-inflammatory cytokines. A decrease in the number of inflammatory macrophages and neutrophils entering the lung tissue and improving lung morphology has been reported [22]. Despite the low implantation rate of stem cells, neutrophil aggregation and pulmonary edema can be prevented [23, 24]. Similarly, bone marrow-derived stem cells can prevent hyperoxia-induced lung injury [19, 25]. Moreover, MSC transplantation can reduce the oxidative stress index [26], and exogenous MSC can retain the production of pulmonary surfactants after hyperoxia injury [27]. Improvements in alveolar and vascular development and a decrease in apoptosis are beneficial to lung growth [28]. In other words, it is not the presence of the cell itself that results in a therapeutic effect, but the components secreted by the cell that repairs lung injury instead. Although the implantation rate of donor cells is low and the cells eventually disappear, exogenous MSCs still have a long-term positive effect [29, 30]. This indicates that the paracrine protection induced by transplanted stem cells may play a key role in tissue repair [29–31]. Importantly MSC paracrine function is tightly regulated by inflammatory signaling pathways, including critical RAP1/NF-5b signaling pathway [32, 33]. Inflammatory cytokines including IL-6 or TNF-*α* not only stimulate MSC to promote tissue repair growth factors and immunomodulatory cytokines [34–36], but they also can promote the efficiency of MSC mitochondrial transfer that can directly alleviate ROS induced-bronchopulmonary damage and improve barrier integrity [37–40]. Mitochondrial damage is widespread in the bronchopulmonary system when exposed to oxidative stress and inflammation. MSC-modulated mitochondrial transfer is a novel mechanism to repair mitochondrial damage in variable tissues, including eye [41] and lung tissues [37, 42].

With the gradual disappearance of donor cells, the intact host tissue protected by stem cell transplantation may continually upregulate various paracrine factors, resulting in lasting beneficial effects. It was found through experiments that after MSC treatment, there was no obvious GFP implantation in the lungs at 1, 3, or 13 weeks, and the implantation rate was 0-5% [19]. We cannot ignore that, given the instability of GFP, the implanted cells will lose their fluorescence. Therefore, the transplanted MSCs may actually exist in the lung structure, but its significant presence cannot be proved.

(3) Immune system interaction

MSCs play an immunomodulatory role through direct cell-to-cell contact as well as prevention of proliferation and function of inflammatory immune cells, including T cells, B cells, natural killer cells, macrophages, monocytes, and dendritic cells [43, 44]. It has been demonstrated that different tissue-derived MSC presents a distinct capacity of immune privilege [45] and potential of immunomodulation [46, 47]. Undifferentiated MSCs express low levels of HLA I and low-level class II. HLA helps avoid recognition by the immune system [48]. Human-induced pluripotent stem cells- (iPSCs-) -MSCs, fetal-MSCs, and bone marrow(BM)-MSCs express little costimulatory molecules [45]. Generally, cells that express major histocompatibility complex (MHC) molecules can either stimulate T cells directly with costimulatory molecules or activate T cells through the cross presentation of MHC antigens of professional antigen-presenting cells (APCs) indirectly. Human or rat MSCs have low immunogenicity and immunosuppressive nature, making them the best choice of clinical application to acquire lower transplantation rejection in the process of allogeneic transplantation. Allo- or xenotransplantation of human or rat MSCs led to a weak immune response in the rat striatum, relative immune cells such as DCs and T*αβ* cells, and no T*γδ*-lymphocytes were expressed in a low level [49]. Studies have shown that the level of HLA I expressed by umbilical cord-derived MSCs is significantly decreased, the concentration of intracellular immunosuppressive molecules increased, and proliferation of T cells inhibited [50, 51].

On the other hand, iPSC-MSCs can not only exert strong immunomodulatory effects [52], but also act directly through the MSC-macrophage interaction: MSC-EXO treatment can attenuate related inflammatory changes and reverse hyperoxia transcriptome changes, reduce the degree of hyperoxia-induced BPD, and improve lung function-lung fibrosis, blood vessels [19]. Remodeling and pulmonary hypertension are suppressed. Furthermore, hyperoxia exposure can change the proangiogenic effect of L-MSC and FGF expression, and decreased expression of CD73 and JAK/STAT indicates decreased immune function [20].

## 3. Experimental Study of MSC Treatment in BPD Animal Model

It is difficult to fundamentally improve the pulmonary function and nervous system prognosis of severe BPD by using the current clinical treatment method. MSC transplantation has become a new hope for BPD treatment, but the specific mechanisms of MSCs in BPD treatment are not very clear. Several animal experimental studies have shown that MSCs can inhibit inflammation, reduce lung injury and pulmonary hypertension, and reduce pulmonary fibrosis [53]. The results of Sutsko et al. showed that the endotracheal administration of MSCs and MSC-conditioned medium in neonatal rats with hyperoxia-induced lung injury suggested that the alveoli continued to improve, inflammation decreased [29], and pulmonary angiogenic factors were upregulated by 28%. In a neonatal rat model, Ahn et al. evaluated the results up to 70 days after birth, they found that the benefits of intratracheal transplantation of MSCs continued over time, and no abnormalities were found in histological examination of various organs [31]. There is growing evidence that MSCs do not implant and reimplant damaged organs, but they function through paracrine activity [16]. This property may explain the multiple functions of MSCs: in addition to their potent anti-inflammatory activity, they also have antifibrosis, antiapoptosis, antioxidant, and angiogenic properties [54].

Currently, the best administration parameters (dosage include) of stem cell therapy for BPD are not clear. Studies have shown that the best dose depends on the injury's location and the route of administration. The best timeframe for stem cell therapy for BPD is also unclear. In animal experiments for stem cell therapy for BPD, the main ways of stem cell transplantation include intratracheal instillation and intravenous and intraperitoneal injections. Sung et al. conducted an animal experiment on lung injury treatment with umbilical cord blood-derived MSCs. They found that the hyperoxia-induced decrease of terminal deoxynucleotidyl transferase deoxyuridine triphosphate nick-end labeling (TUNEL)-positive cells in the alveolar damage index in the intratracheal injection group was more evident than that in the intravenous injection group. Therefore, the authors believe that intratracheal injection is more effective than intravenous infusion [28]. In Pierro et al.'s study, after intratracheal injection of perivascular cells and MSCs, the follow-up results of neonatal rats showed that although the amount of cell implantation was low, lung repair and function improved over a long period due to the paracrine effects of MSCs [30]. Van Haaften et al. showed that in the neonatal rat model of BPD, MSC transplantation on the 4th day after birth could significantly reduce lung injury. For clinical treatment, early identification of BPD and early stem cell transplantation may be more effective [19]. A meta-analysis by Augustine et al. showed that the therapeutic effect of MSCs was significantly better than that of the control group in all intraperitoneal, endotracheal, and intravenous subgroups. Intravenous administration was substantially better than intratracheal administration. Bone marrow MSCs were more commonly used than umbilical cord stem cells. There was no significant statistical difference among low, middle, and high doses of MSCs. Compared with the control group, the effect of MSC treatment was beneficial regardless of treatment and evaluation timing [55]. We summarized the representative animal studies, these animal experiments have adopted the same model hyperoxia-induced BPD in newborn rats, and these results represented the advantages and huge potential of the MSC application. The characteristics of the animal experiments are shown in Table 1. Through these reports, even though the management is quite different from the dose or timing or ways of transplantation, MSC administration could decrease both mean linear intercept and alveolar volume, which indicates intervention is benefit for lung alveolarization. And most experiments are strong evidence for that the paracrine activities play important roles in this process.

## 4. Study of MSC Treatment in Neonatal BPD

Presently, the clinical stem cell therapy research for BPD is mainly focused on umbilical cord blood-derived MSCs, umbilical cord-derived MSCs, and bone marrow MSCs. Human umbilical cord tissue is considered the most attractive source of MSCs because it is readily available. Compared with other sources of MSCs, it shows less antigenicity, more significant cell proliferation, and potentially superior repair potential [56–58].

Search for ongoing studies on the clinical intervention of mesenchymal stem cells in BPD in the database provided by the U.S. National Library of Medicine (https://clinicaltrials.gov/), as shown in Table 2.

Chang et al. used human umbilical cord blood-derived MSCs in an experimental group through endotracheal therapy. The children who received stem cell therapy did not receive oxygen therapy after discharge, while 22% of the control group children needed home oxygen therapy after discharge. The severity of BPD was lower in patients with MSC transplantation. There was no difference in the incidence of other adverse outcomes between the control group and patients with MSC transplantation [57]. Currently, MSC transplantation for BPD is still in the clinical research stage, and the safety of MSC transplantation is the issue of concern for researchers and clinicians. The clinical trials of Ahn et al. showed that intratracheal transplantation of MSC was safe, and no obvious adverse reactions were found during the follow-up to the corrected age of 2 years old [59].

Powell and Silvestri evaluated the safety of intratracheal administration of a single dose of human umbilical cord blood MSCs in 12 ELBW infants with BPD at 28 weeks of gestation within 5–14 days after birth, suggesting that the treatment is well tolerated, seems safe, and feasible [60]. The characteristics of the clinical trials are summarized in Table 3.

As stem cells can regenerate and differentiate, the biggest concern is whether the possibility of stem cells differentiating into tumor cells can be ruled out. The possible reasons are as follows: (1) The malignant transformation of bone marrow mesenchymal stem cells may be due to the potential risk of further malignant development and transformation of bone marrow mesenchymal stem cells in vitro, which may be the immunosuppressive environment created by these cells. (2) Bone marrow mesenchymal stem cells. Its immunosuppressive effect may cause the growth of tumor cells in the patient's existing malignant cells [61]. In 2010, De la Fuente et al. [62] published articles in Cancer Research magazine, reminding researchers that MSCs may spontaneously transform into tumor cells. Since then, many researchers have carried out a large number of related animal experiments.

However, after MSC treatment, subjects in this phase I clinical trial were followed up for 2 years to study its long-term safety and effects on growth, respiratory symptoms, and neurodevelopmental outcomes, without any adverse consequences related to transplantation, including tumorigenicity [61]. According to some preclinical studies and meta-analysis related to MSC, there is no evidence of adverse events or tumor formation including tumorigenicity until 60 months after treatment. The current mainstream view is that MSCs will not change spontaneously in malignant transformation [61].

## 5. Current Challenges in MSC Treatment

1. Heterogeneity of MSCs

MSCs can be isolated not only from adult tissues, including bon barrow, fat tissues, and cord tissues. Such adult tissue-derived MSCs are highly heterogeneous. These MSCs derived from different donors often display batch-to-batch variations, the variable of stem cell senescence, and proliferative potency that affects accuracy in MSC studies. MSCs can also be derived from the same parental pluripotent stem cells that overcome many disadvantages of adult MSCs, more homogeneous with less batch-to-batch variations in MSC quality, stem cell senescence, and limited proliferative potency. Human PSC-derived MSCs possess higher proliferative potential [63] and display strong immunomodulation. Stem cells from different tissue sources also have heterogeneity [64]. Stem cell heterogeneity is mainly reflected in their proliferation ability, growth characteristics, biological characteristics such as migration ability, immune regulation ability, and the type of cytokines secreted. The multidifferentiation efficiency of MSC will have a positive impact on the pathogenesis of multifactorial disease BPD. Therefore, it is necessary to select the best indication according to the heterogeneity of MSCs. MSC can trigger an immediate prethrombotic inflammatory response in the blood. Compared with endothelial cells, MSCs exposed to whole blood in vitro have been found to cause immediate inflammation. In addition, this effect demonstrates the variability between donors and becomes more effective as the number of MSC delivery increases [65]. Still, allotransplantation risks are present, although phase I clinical trials do not show any short-term side effects [57, 66, 67]. Presently, the differentiation of MSCs and the loss of related functions cannot be monitored by quality control experiments [68]. Most recently, GMP-grade MSCs derived from hiPSCs have been used in refractory graft-versus-host-disease (GVHD) in clinical trials [69]. PSC-MSC may provide another putative cellular source overcome many limitations of adult MSC.

(2) The biological efficacy and teratogenicity of MSCs

Currently, studies on the biological effectiveness of MSCs mainly evaluate the level of secreted cytokines, hematopoiesis promotion, neovascularization, immunosuppression, antifibrosis, and so on. It is yet to be determined whether embryonic stem cells (ESCs) can be useful as a treatment for lung injury. In vitro, mouse-derived ESCs have been shown to differentiate into type II alveolar epithelial cells (AEC-IIs) [70]. When cocultured with fetal lungs, they form pseudoglandular epithelial structures and express surfactant protein C (SP-C), which is a sign of functional AEC-IIs [71]. The expression of thyroid transcription factor (TTF-1) is a marker of early lung development. The expression of aquaporin 5 and SP-C are the signs of AEC-Is and AEC-IIs in alveolar epithelial cells, respectively. During the perinatal period, ESC-derived AECs can rescue fetal lung explants from pulmonary hypoplasia and vascular rupture in vitro [72]. It is necessary to screen MSC-related markers through the laboratory and then carry out verification experiments and quantitative determination of biological activity, to lay a foundation for the application and promotion of MSCs which can affect the proliferation and function of immune cells, including dendritic cells, NK cells, and T and B lymphocytes [43, 73, 74]. It should be noted that tumor formation has not yet been verified after MSC treatment in vivo [59, 61, 66], but it does not mean that MSCs have no potential for teratogenic and carcinogenic. According to reports, chromosomal abnormalities rarely occur in primary cells [75]. Different from embryonic machinery cells which may lead to teratoma formation after implantation of ESCs in vivo [76] or induced pluripotent stromal cells, MSCs are adult stromal cells, which have limited expansion ability, and heterogeneous cell populations composed of cells with different population multiples, which, therefore, are more inclined to chromosomal aberrations. At present, it is impossible to predict whether genomic instability during MSC amplification is related to changes in the in vitro environment during the passage and whether cell proliferation will lead to heterogeneous subsets. Still, the possible supporting effect of MSCs on the matrix of tumor cells [77] and the carcinogenic potential of bronchoalveolar stem cells [78, 79] worry researchers. Besides, another problem is the possibility of immune rejection of the transplanted stem cells.

(3) The problem of MSC derivatives

In addition to MSCs, derivatives of MSCs such as exocrine, microRNA, and stem cell factors also have therapeutic effects. If the therapeutic effects of MSCs are similar to those of stem cell derivatives, then the idea of using stem cell derivatives to replace stem cell therapy can be considered. To overcome these difficulties, the supernatant or exocrine derived from MSCs has become a promising substitute, and promising results have been obtained in previous animal experiments [80].

(4) The ethics of MSC transplantation

MSC transplantation for BPD treatment requires ethical review and evaluation before clinical trials. The differentiation and related functional loss of MSCs during amplification cannot be monitored by quality control analysis, but cell culture without animal components is a promising research direction [68]. Moreover, conceptually, the expansion of MSCs should be considered to overcome the risks and ethical concerns of allotransplantation. And the clinical trials published to date show the effectiveness of MSC transplantation in BPD have been largely exaggerated—neither of these trials are meant to show efficacy. MSCs, unlike ESCs, are not reported to induce tumors. Ethical considerations are not a limitation, once donor consent is obtained.

(5) To formulate the clinical plan of MSC transplantation for the BPD treatment

Although there are many animal experimental studies of MSC transplantation in the treatment of BPD, there is no unified clinical scheme of MSC transplantation for neonatal BPD. The predictive model, treatment time window, and optimal BPD dose need to be improved in clinical trials in the future. MSCs have entered the clinical research stage, and the preliminary results have shown the effectiveness and safety of MSC transplantation in BPD treatment. Many neonatal medical centers have applied for relevant clinical trials. Simultaneously, the clinical research of stem cells in the treatment of other neonatal diseases is also being carried out gradually.

## 6. Conclusion

As the survival rate of premature infants increases, the incidence of BPD continues to increase. Early prevention, early diagnosis, and early treatment are particularly important, but there are no safe and effective prevention and treatment measures. In recent years, stem cell therapy has brought new hope to the prevention and treatment of BPD. In summary, MSC therapy has been used in clinical studies of many diseases and has proven to have advantages such as reduced inflammation, lung injury, and fibrosis. However, more multicenter, large-scale, prospective clinical randomized controlled trials are needed to strongly advocate for MSC therapy as an option for BPD treatment.

## Figures and Tables

**Table 1:** Summary of study characteristics extracted from induced animal experiments.

Author (year)	Oxygen concentration	Dose (per animal), time (hours), method	Time of assessment	Outcomes
van Haaften (2009)	95%	(1) 1 × 10^5^ BMSC, P4, IT (2) BMSC 1 × 10^5^, P14, IT	P21, P4	Alveolarization, lung angiogenesis, PH, exercise capacity, survival rate
Aslam (2009)	75%	(1) 5 × 10^4^ cells P4, IV (2) BMSC-CM 50 *μ*l, P4, IV	P5/P14	Alveolarization, lung inflammation, PH, vascular injury
Chang (2009)	95%	(1) 2 × 10^6^ cells, P5, IT (2) 5 × 10^5^ cells, P5, IP	P14	Weight, survival, alveolarization, apoptosis, lung inflammation, and fibrosis
Chang (2011)	95%	(1) 5 × 10^3^ cells (2) 5 × 10^4^ cells (3) 5 × 10^5^ cells, P5, IT	P14	Weight, survival, alveolarization, apoptosis, lung inflammation, MPO activity, lung fibrosis, oxidative stress
Chang (2013)	90%	5 × 10^5^ cells P3/P10/P3+P10, IT	P1, P3, P5, P7, P10 P14, P21	Weight, survival, alveolarization, apoptosis, lung inflammation, fibrosis, oxidative stress
Zhang (2012)	95%	1 × 10^5^ cells, P10, IV	P13, P17, P24	Weight gain, lung inflammation, alveolarization, tissue cytokine
Waszak (2012)	95%	1 ml/kg, P0-P20, IP	P21	Alveolarization, pH, PA remodeling
Sutsko (2013)	90%	(1) 2 × 10^6^ cells, P9, IT (2) 0.05 ml MSC-CM, IT	P16, P30, P100	Alveolarization, lung angiogenesis and inflammation, PH
Pierro (2013)	95%	(1) 3 × 10^5^ MSCs, P4, IT (2) 6 × 10^5^ MSCs, P14, IT (3) 7 *μ*l/g CdM, P4-P21, IP (4) 7 *μ*l/g CdM, P14-P28, IP	P22, P35, P6 months	Alveolarization, PA remodeling, PH, lung angiogenesis, function, exercise capacity
Ahn (2013)	90%	5 × 10^5^ cells, P5, IT	P70	Alveolarization, lung inflammation, angiogenesis, safety, weight, survival rate
Sung (2015)	90%	(1) 5 × 10^5^ cells P5, IT (2) 2 × 10^6^ cells P5, IV	P14	Alveolarization, lung inflammation Cell death, fibrosis
Ahn (2015)	90%	(1) hUB-MSC 5 × 10^5^ cells (2) AT MSC 5 × 10^5^ cells (3) hUB-MNC 5 × 10^5^ cells; P5, IT	P7, P14	Alveolarization, lung inflammation, angiogenesis
Gulasi (2016)	85-95%	(1) 1 × 10^5^ cells (2) Culture medium 25 *μ*l (3) Remaining medium 25 *μ*l, P11, IT	P10, P60	Lung/body weight, alveolarization, lung fibrosis, inflammation

Abbreviations: P: postnatal; IV: intravenous; IT: intratracheal; IP: intraperitoneal; IN: intranasal; PASMC: pulmonary artery smooth muscle cell; CM: conditioned media; MSC: mesenchymal stem/stromal cell; PH: pulmonary hypertension; PA: pulmonary artery; BASC: bronchoalveolar stem cell; MPO: myeloperoxidase; UCB: umbilical cord blood; hUCT: human umbilical cord tissue; AT: adipose tissue; KGF: keratinocyte growth factor; CdM: chemically defined media; P38MAPK: mitogen-activated protein kinase; ERK: extracellular signal-regulated kinase.

**Table 2:** Clinical study of registered MSC transplantation in the treatment of neonatal BPD.

NCT number	Country	Status	Phase	Enrollment	Cell type	Start date	Completion date
NCT04062136	Vietnam	Recruiting	1	10	hUC-MSC	1-Mar-19	30-Nov-20
NCT03873506	China	Recruiting	1	30	hUC-MSC	1-Jul-18	31-Dec-20
NCT03645525	China	Recruiting	1|2	180	hUC-MSC	1-Dec-19	1-Apr-21
NCT03601416	China	Not yet recruiting	2	57	MSC	1-Jul-19	31-Dec-21
NCT03378063	China	Recruiting	Early 1	100	hMSC	1-Nov-17	30-Dec-22
NCT03631420	China	Recruiting	1	9	hUC-MSC	26-Oct-18	31-Oct-22
NCT04255147	Canada	Not yet recruiting	1	9	hUCT-MSC	Jan-21	Dec-35
NCT02443961	Spain	Recruiting	1	10	MSC	2-Apr-19	Apr-25
NCT03774537	China	Recruiting	1|2	20	hUC-MSC	1-Mar-19	31-Dec-21
NCT01632475	South Korea	Active, not recruiting	Unclear	9	hUC-MSC	Sep-11	Sep-26
NCT03392467	South Korea	Recruiting	2	60	hUC-MSC	13-Aug-18	Jul-21
NCT04003857	South Korea	Recruiting	2	60	hUC-MSC	5-Jul-19	30-Jun-27
NCT03558334	China	Recruiting	1	12	hMSC	28-Jun-18	30-Jun-22

**Table 3:** Summary of study characteristics extracted from included clinical trials.

Author	Number	Mean birth weight	Mean time after birth	Dose of MSC	Method	Stem cell source	Aim
Chang (2014)	9	630-1030 g	7-14 d	(1) Low dose: 1 × 10^7^ cells/kg (2) High dose: 2 × 10^7^ cells/kg	IT	Allograft transplantation	Assess the safety and feasibility of allogeneic hUC-MSC transplantation in preterm infants
Powell (2019)	12	500-1000 g	No surfactant within 24 hours	(1) Low dose: 1 × 107 cells/kg (2) High dose: 2 × 107 cells/kg	IT	Allograft transplantation	Assess the safety of intratracheal administration of hUC-MSCs into premature infant patients at high risk for BPD

## Data Availability

The data used to support the findings of this study are available from the corresponding author upon request.

## References

[1] Owen L., Manley B., Davis P., Doyle L. (2017). The evolution of modern respiratory care for preterm infants. *The Lancet*.

[2] Thekkeveedu R., Cuevas Guaman M., Shivanna B. (2017). Bronchopulmonary dysplasia: a review of pathogenesis and pathophysiology. *Respiratory Medicine*.

[3] Kondrikov D., Caldwell R., Dong Z., Su Y. (2011). Reactive oxygen species-dependent RhoA activation mediates collagen synthesis in hyperoxic lung fibrosis. *Free Radical Biology & Medicine*.

[4] Bhandari A., McGrath-Morrow S. (2013). Long-term pulmonary outcomes of patients with bronchopulmonary dysplasia. *Seminars in Perinatology*.

[5] Timmers L., Lim S. K., Arslan F. (2007). Reduction of myocardial infarct size by human mesenchymal stem cell conditioned medium. *Stem Cell Research*.

[6] Doyle L. W., Ehrenkranz R. A., Halliday H. L. (2014). Early (<8 days) postnatal corticosteroids for preventing chronic lung disease in preterm infants. *Cochrane Database of Systematic Reviews*.

[7] Darlow B. A., Graham P. (2007). Vitamin A supplementation for preventing morbidity and mortality in very low birthweight infants (Cochrane review). *Cochrane database of systematic reviews*.

[8] Hass R., Kasper C., Bohm S., Jacobs R. (2011). Different populations and sources of human mesenchymal stem cells (MSC): a comparison of adult and neonatal tissue-derived MSC. *Cell Communication and Signaling*.

[9] Dominici M. L., Le Blanc K., Mueller I. (2006). Minimal criteria for defining multipotent mesenchymal stromal cells. *The International Society for Cellular Therapy position statement.*.

[10] Collins J., Thébaud B. (2013). Lung Mesenchymal Stromal Cells in Development and Disease: To Serve and Protect?. *Antioxidants & redox signaling*.

[11] Chamberlain G., Fox J., Ashton B., Middleton J. (2007). Concise review: mesenchymal stem cells: their phenotype, differentiation capacity, immunological features, and potential for homing. *Stem Cells*.

[12] Honczarenko M., Le YI S. M., Ghiran I., Glodek A. M., Silberstein L. E. (2006). Human bone marrow stromal cells express a distinct set of biologically functional chemokine receptors. *Stem cells*.

[13] Ren G., Chen X., Dong F. (2012). Concise review: mesenchymal stem cells and translational medicine: emerging issues. *Stem Cells Translational Medicine*.

[14] Sueblinvong V., Loi R., Eisenhauer P. L. (2008). Derivation of lung epithelium from human cord blood–derived mesenchymal stem cells. *American Journal of Respiratory and Critical Care Medicine*.

[15] Murray I. R., West C. C., Hardy W. R. (2014). Natural history of mesenchymal stem cells, from vessel walls to culture vessels. *Cellular and Molecular Life Sciences*.

[16] Fung M., Thébaud B. (2013). Stem cell-based therapy for neonatal lung disease: it is in the juice. *Pediatric Research*.

[17] Tropea K. A., Leder E., Aslam M. (2012). Bronchioalveolar stem cells increase after mesenchymal stromal cell treatment in a mouse model of bronchopulmonary dysplasia. *American Journal of Physiology. Lung Cellular and Molecular Physiology*.

[18] Lee J. W., Fang X., Krasnodembskaya A., Howard J. P., Matthay M. A. (2011). Concise review: mesenchymal stem cells for acute lung injury: role of paracrine soluble factors. *Stem Cells*.

[19] van Haaften T., Byrne R., Bonnet S. (2009). Airway delivery of mesenchymal stem cells prevents arrested alveolar growth in neonatal lung injury in rats. *American Journal of Respiratory and Critical Care Medicine*.

[20] Aslam M., Baveja R., Liang O. D. (2009). Bone marrow stromal cells attenuate lung injury in a murine model of neonatal chronic lung disease. *American Journal of Respiratory and Critical Care Medicine*.

[21] Friedenstein A., Chailakhyan R., Latsinik N., Panasyuk A., Keiliss-Borok I. (1974). Stromal cells responsible for transferring the microenvironment of the hemopoietic tissues. Cloning in vitro and retransplantation in vivo. *Transplantation*.

[22] Chou H.-C., Li Y.-T., Chen C.-M. (2016). Human mesenchymal stem cells attenuate experimental bronchopulmonary dysplasia induced by perinatal inflammation and hyperoxia. *American Journal of Translational Research*.

[23] Iyer S., Co C., Rojas M. (2009). Mesenchymal stem cells and inflammatory lung diseases. *Panminerva Medica*.

[24] Mei S. H., SD M. C., Deng Y., Parker C. H., Liles W. C., Stewart D. J. (2007). Prevention of LPS-induced acute lung injury in mice by mesenchymal stem cells overexpressing angiopoietin 1. *PLoS Medicine*.

[25] Balasubramaniam V., Ryan S. L., Seedorf G. J. (2010). Bone marrow-derived angiogenic cells restore lung alveolar and vascular structure after neonatal hyperoxia in infant mice. *American Journal of Physiology. Lung Cellular and Molecular Physiology*.

[26] Chang Y. S., Choi S. J., Ahn S. Y. (2013). Timing of umbilical cord blood derived mesenchymal stem cells transplantation determines therapeutic efficacy in the neonatal hyperoxic lung injury. *PLoS One*.

[27] Chang Y. S., Oh W., Choi S. J. (2009). Human umbilical cord blood-derived mesenchymal stem cells attenuate hyperoxia-induced lung injury in neonatal rats. *Cell Transplantation*.

[28] Sung D. K., Chang Y. S., Ahn S. Y. (2015). Optimal route for human umbilical cord blood-derived mesenchymal stem cell transplantation to protect against neonatal hyperoxic lung injury: gene expression profiles and histopathology. *PLoS One*.

[29] Sutsko R. P., Young K. C., Ribeiro A. (2013). Long-term reparative effects of mesenchymal stem cell therapy following neonatal hyperoxia-induced lung injury. *Pediatric Research*.

[30] Pierro M., Ionescu L., Montemurro T. (2013). Short-term, long-term and paracrine effect of human umbilical cord-derived stem cells in lung injury prevention and repair in experimental bronchopulmonary dysplasia. *Thorax*.

[31] Ahn S. Y., Chang Y. S., Kim S. Y. (2013). Long-term (postnatal day 70) outcome and safety of intratracheal transplantation of human umbilical cord blood-derived mesenchymal stem cells in neonatal hyperoxic lung injury. *Yonsei Medical Journal*.

[32] Ding Y., Liang X., Zhang Y. (2018). Rap1 deficiency-provoked paracrine dysfunction impairs immunosuppressive potency of mesenchymal stem cells in allograft rejection of heart transplantation. *Cell Death & Disease*.

[33] Zhang Y., Chiu S., Liang X. (2015). Rap1-mediated nuclear factor-kappaB (NF-kappaB) activity regulates the paracrine capacity of mesenchymal stem cells in heart repair following infarction. *Cell death discovery*.

[34] Philipp D., Suhr L., Wahlers T., Choi Y. H., Paunel-Görgülü A. (2018). Preconditioning of bone marrow-derived mesenchymal stem cells highly strengthens their potential to promote IL-6-dependent M2b polarization. *Stem Cell Research & Therapy*.

[35] Cho D. I., Kim M. R., Jeong H. Y. (2014). Mesenchymal stem cells reciprocally regulate the M1/M2 balance in mouse bone marrow-derived macrophages. *Experimental & Molecular Medicine*.

[36] Jaimes Y., Naaldijk Y., Wenk K., Leovsky C., Emmrich F. (2017). Mesenchymal stem cell-derived microvesicles modulate lipopolysaccharides-induced inflammatory responses to microglia cells. *Stem Cells*.

[37] Li X., Michaeloudes C., Zhang Y. (2018). Mesenchymal stem cells alleviate oxidative stress-induced mitochondrial dysfunction in the airways. *Journal of Allergy and Clinical Immunology.*.

[38] Zhao M., Liu S., Wang C. (2021). Mesenchymal stem cell-derived extracellular vesicles attenuate mitochondrial damage and inflammation by stabilizing mitochondrial DNA. *ACS Nano*.

[39] Silva J. D., Su Y., Calfee C. S. (2020). MSC extracellular vesicles rescue mitochondrial dysfunction and improve barrier integrity in clinically relevant models of ARDS. *The European Respiratory Journal*.

[40] Yao Y., Fan X. L., Jiang D. (2018). Connexin 43-mediated mitochondrial transfer of iPSC-MSCs alleviates asthma inflammation. *Stem Cell Reports*.

[41] Jiang D., Xiong G., Feng H. (2019). Donation of mitochondria by iPSC-derived mesenchymal stem cells protects retinal ganglion cells against mitochondrial complex I defect-induced degeneration. *Theranostics*.

[42] Li X., Zhang Y., Yeung S. C. (2014). Mitochondrial transfer of induced pluripotent stem cell-derived mesenchymal stem cells to airway epithelial cells attenuates cigarette smoke-induced damage. *American Journal of Respiratory Cell and Molecular Biology*.

[43] Singer N. G., Caplan A. I. (2011). Mesenchymal stem cells: mechanisms of inflammation. *Annual Review of Pathology*.

[44] Gebler A., Zabel O., Seliger B. (2012). The immunomodulatory capacity of mesenchymal stem cells. *Trends in Molecular Medicine*.

[45] Sun Y. Q., Zhang Y., Li X. (2015). Insensitivity of human iPS cells-derived mesenchymal stem cells to interferon-gamma-induced HLA expression potentiates repair efficiency of hind limb ischemia in immune humanized NOD Scid gamma mice. *Stem Cells*.

[46] Sun Y. Q., Deng M. X., He J. (2012). Human pluripotent stem cell-derived mesenchymal stem cells prevent allergic airway inflammation in mice. *Stem Cells*.

[47] Fu Q. L., Chow Y. Y., Sun S. J. (2012). Mesenchymal stem cells derived from human induced pluripotent stem cells modulate T-cell phenotypes in allergic rhinitis. *Allergy*.

[48] Le Blanc K., Tammik C., Rosendahl K., Zetterberg E., Ringdén O. (2003). HLA expression and immunologic properties of differentiated and undifferentiated mesenchymal stem cells. *Experimental Hematology*.

[49] Rossignol J., Boyer C., Thinard R. (2009). Mesenchymal stem cells induce a weak immune response in the rat striatum after allo or xenotransplantation. *Journal of Cellular and Molecular Medicine*.

[50] Deuse T., Stubbendorff M., Tang-Quan K. (2011). Immunogenicity and immunomodulatory properties of umbilical cord lining mesenchymal stem cells. *Cell Transplantation*.

[51] Najar M., Raicevic G., Boufker H. I. (2010). Mesenchymal stromal cells use PGE2 to modulate activation and proliferation of lymphocyte subsets: combined comparison of adipose tissue, Wharton's Jelly and bone marrow sources. *Cellular immunology*.

[52] Wang L. T., Jiang S. S., Ting C. H. (2018). Differentiation of mesenchymal stem cells from human induced pluripotent stem cells results in downregulation of c-Myc and DNA replication pathways with immunomodulation toward CD4 and CD8 cells. *Stem Cells*.

[53] Wecht S., Rojas M. (2016). Mesenchymal stem cells in the treatment of chronic lung disease: mesenchymal stem cells and lung injury. *Respirology*.

[54] Thébaud B., Kourembanas S. (2017). Can we cure bronchopulmonary dysplasia?. *The Journal of pediatrics*.

[55] Augustine S., Avey M. T., Harrison B. (2017). Mesenchymal stromal cell therapy in bronchopulmonary dysplasia: systematic review and meta-analysis of preclinical studies. *Stem Cells Translational Medicine*.

[56] Hsieh J. Y., Wang H. W., Chang S. J. (2013). Mesenchymal stem cells from human umbilical cord express preferentially secreted factors related to neuroprotection, neurogenesis, and angiogenesis. *PLoS One*.

[57] Chang Y. S., Ahn S. Y., Yoo H. S. (2014). Mesenchymal stem cells for bronchopulmonary dysplasia: phase 1 dose-escalation clinical trial. *The Journal of pediatrics*.

[58] Yannarelli G., Dayan V., Pacienza N., Lee C. J., Medin J., Keating A. (2013). Human umbilical cord perivascular cells exhibit enhanced cardiomyocyte reprogramming and cardiac function after experimental acute myocardial infarction. *Cell Transplantation*.

[59] Ahn S. Y., Chang Y. S., Kim J. H., Sung S. I., Park W. S. (2017). Two-year follow-up outcomes of premature infants enrolled in the phase I trial of mesenchymal stem cells transplantation for bronchopulmonary dysplasia. *The Journal of Pediatrics*.

[60] Powell S. B., Silvestri J. M. (2019). Safety of intratracheal administration of human umbilical cord blood derived mesenchymal stromal cells in extremely low birth weight preterm infants. *The Journal of Pediatrics*.

[61] Barkholt L., Flory E., Jekerle V. (2013). Risk of tumorigenicity in mesenchymal stromal cell-based therapies--bridging scientific observations and regulatory viewpoints. *Cytotherapy*.

[62] de la Fuente R., Bernad A., Garcia-Castro J., Martin M. C., Cigudosa J. C. (2010). Retraction: spontaneous human adult stem cell transformation. *Cancer Research*.

[63] Zhang J., Chan Y. C., Ho J. C. Y., Siu C. W., Lian Q., Tse H. F. (2012). Regulation of cell proliferation of human induced pluripotent stem cell-derived mesenchymal stem cells via ether-à-go-go 1 (hEAG1) potassium channel. *American Journal of Physiology. Cell Physiology*.

[64] Wagner W. (2010). Senescence is heterogeneous in mesenchymal stromal cells: kaleidoscopes for cellular aging. *Cell cycle*.

[65] Moll G., Rasmusson-Duprez I., von Bahr L. (2012). Are therapeutic human mesenchymal stromal cells compatible with human blood?. *Stem Cells*.

[66] Cotten C. M., Murtha A. P., Goldberg R. N. (2014). Feasibility of autologous cord blood cells for infants with hypoxic-ischemic encephalopathy. *Journal of Pediatrics*.

[67] Arutyunyan I., Elchaninov A., Makarov A., Fatkhudinov T. (2016). Umbilical cord as prospective source for mesenchymal stem cell-based therapy. *Stem Cells International*.

[68] Blázquez Prunera A., Almeida C., Barbosa M. (2017). Human bone marrow mesenchymal stem/stromal cells preserve their immunomodulatory and chemotactic properties when expanded in a human plasma derived xeno-free medium. *Stem Cells International*.

[69] Bloor A. J. C., Patel A., Griffin J. E. (2020). Production, safety and efficacy of iPSC-derived mesenchymal stromal cells in acute steroid-resistant graft versus host disease: a phase I, multicenter, open-label, dose-escalation study. *Nature Medicine*.

[70] Denham M., Cole T., Mollard R. (2006). Embryonic stem cells form glandular structures and express surfactant protein C following culture with dissociated fetal respiratory tissue. *American Journal of Physiology. Lung Cellular and Molecular Physiology*.

[71] Ali N. N., Edgar A. J., Samadikuchaksaraei A. (2002). Derivation of type II alveolar epithelial cells from murine embryonic stem cells. *Tissue Engineering*.

[72] Roszell B., Seaton A., Fong G.-H., Finck C. (2009). Cell-based therapy improves cell viability and increases airway size in an explant model. *Experimental Lung Research*.

[73] Le Blanc K., Davies L. C. (2015). Mesenchymal stromal cells and the innate immune response. *Immunology Letters*.

[74] Zahari W., Hashim S., Yusof M. (2017). Immunomodulatory effect of cytokines in the differentiation of mesenchymal stem cells: a review. *Current Stem Cell Research & Therapy*.

[75] Prockop D. J., Brenner M., Fibbe W. E. (2010). Defining the risks of mesenchymal stromal cell therapy. *Cytotherapy*.

[76] Blum B., Benvenisty N. (2008). The tumorigenicity of human embryonic stem cells. *Advances in Cancer Research*.

[77] Kidd S., Spaeth E., Klopp A., Andreeff M., Hall B., Marini F. (2008). The (in) auspicious role of mesenchymal stromal cells in cancer: be it friend or foe. *Cytotherapy*.

[78] Dovey J. S., Zacharek S. J., Kim C. F., Lees J. A. (2008). Bmi1 is critical for lung tumorigenesis and bronchioalveolar stem cell expansion. *Proceedings Of The National Academy Of Sciences*.

[79] Kim C. F., Jackson E. L., Woolfenden A. E. (2005). Identification of bronchioalveolar stem cells in normal lung and lung cancer. *Cell*.

[80] Lee C., Mitsialis S. A., Aslam M. (2012). Exosomes mediate the cytoprotective action of mesenchymal stromal cells on hypoxia-induced pulmonary hypertension. *Circulation*.

